# Between the sheets

**DOI:** 10.5195/jmla.2017.195

**Published:** 2017-10-01

**Authors:** Stephen J. Greenberg

We are, or at least most of us are, librarians. We tend collections that are still mostly made up of physical items on a series of shelves (for the purposes of this discussion, I am considering physical journals, bound or unbound, to be books. Humor me, please). Some of our collections are reaching a tipping point where the electronic resources we have will outweigh (intellectually, at least) the paper. Some of us are there already; many more will be there soon.

Books on paper, the physical objects, have reached a very odd place in our consciousness. Readers are increasingly offered books (or at least texts—there is a difference) in a bewildering array of electronic alternatives. We are told that books on paper are dead. At the same time, the latest Harry Potter story sold two million print copies in the United States and Canada in its first two days of availability.

Oddly, at no time in the history of printing has it been easier to produce a physical book. Websites such as Blurb.com and Lulu.com allow authors to self-publish books in a variety of formats, with drag-and-drop interfaces, fast turnover times, and remarkably good results. Self-publishing is hardly new, but modern technology allows extremely small press-runs, even as small as a single copy. I recently self-published a book of my (nonmedical, nonhistorical) photographs through one such website and was quite gratified by the results. The original press run was to be eight copies; due to a glitch in the layout software that the web company provided and their conscientious customer service folks, that number swelled to sixteen. If you are a rare book cataloguer, I would say there are two states of a single edition, since the differences between the first run and the second run, while real, are so small as to be almost laughable.

This is NOT how it was. During the hand-press era of Western printing (roughly 1450 to the early 19th century), books were laboriously created with handset type, on single sheets of handmade paper, with clumsy wooden (eventually iron) presses operated by human muscle. Although there were exceptions in special cases, a press run needed to be about 1,500 copies for anyone (author, printer, or publisher) to make any money. The large sheets of paper were printed on one side, allowed to dry, printed on the other side (the technical term was “perfecting”), allowed to dry again, folded, gathered, stitched, and (possibly) bound for sale. None of this work was mechanized until well into the 19th century.

It is exceedingly rare to see an original hand-press book in an intermediate stage of production, what is technically called a book in sheets. But in the collections of the National Library of Medicine (NLM), there exists just such a book: printed sheets from 1610, never folded, never bound, never quite finished. It is titled *Prognosticatio Eximii Doctrois Theophrasti Paracelsi*, which translates (roughly) from the Latin as predictions or prophecies from the famous doctor, pharmacologist, alchemist, astrologer, and much else, Philippus Aureolus Theophrastus Bombastus von Hohenheim (1493–1541), normally just known as Paracelsus. His work in toxicology, particularly involving the medical uses of mercury, was groundbreaking. His philosophical and mystical work, particularly as shown in this book, which was reprinted many times, simply demonstrates how low the boundaries were between science and medicine at the time. Paracelsus was a seriously fun guy, travelling widely through Europe, experimenting, prophesying, writing, picking fights, and getting chased out of various cities. He died in Strasbourg at the age of forty-seven, possibly in a bar fight.

**Figure 1:**
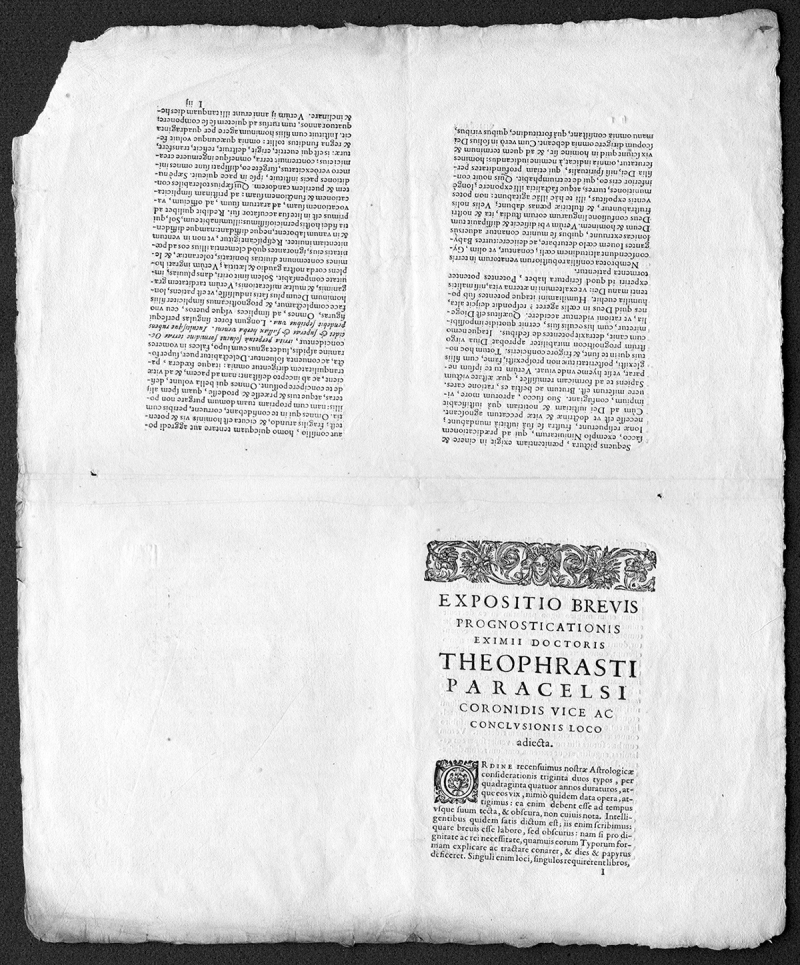
Complete sheet

The *Prognosticatio* consists of 10 sheets, roughly 47×38 centimeters each, of handmade paper just as it would have emerged from the press after the second side was printed. Four pages of text and illustrations are visible on each side of the sheet, their orientation depending on how the sheet was to be folded. In this case, the sheet was to be folded twice, to provide a quarto-format gathering of 8 pages. Had the sheet shown 2 pages per side, designed to be folded once, it would have been a folio; had it been printed with 8 pages showing, designed to be folded 3 times, it would have been an octavo. It is worth remembering that the terms folio, quarto, octavo, and so on refer to how the sheet was to be folded after printing. They have no direct bearing on the size of the book itself in the hand-press era. Figure 1 shows a complete sheet and illustrates how the sheet would need to be folded to result in the proper orientation and order.

Often, printed sheets would have additional letters called “signatures” in the lower margins to help the bookbinder place the gatherings in the correct order for sewing. This would be especially important when gatherings were to be nested inside each other, a common type of book structure. The main advantage of such nesting was that it needed less sewing to put the book together; the disadvantage was that it made the job of typesetting more complicated, as the text needed to be oriented just right over multiple settings of type and sheets. Both systems were commonly used. In the *Prognosticatio,* it is clear, based on the signature letters, that nesting was not used. The letters also indicated that the book was complete, as there were no gaps in their order.

**Figure 2:**
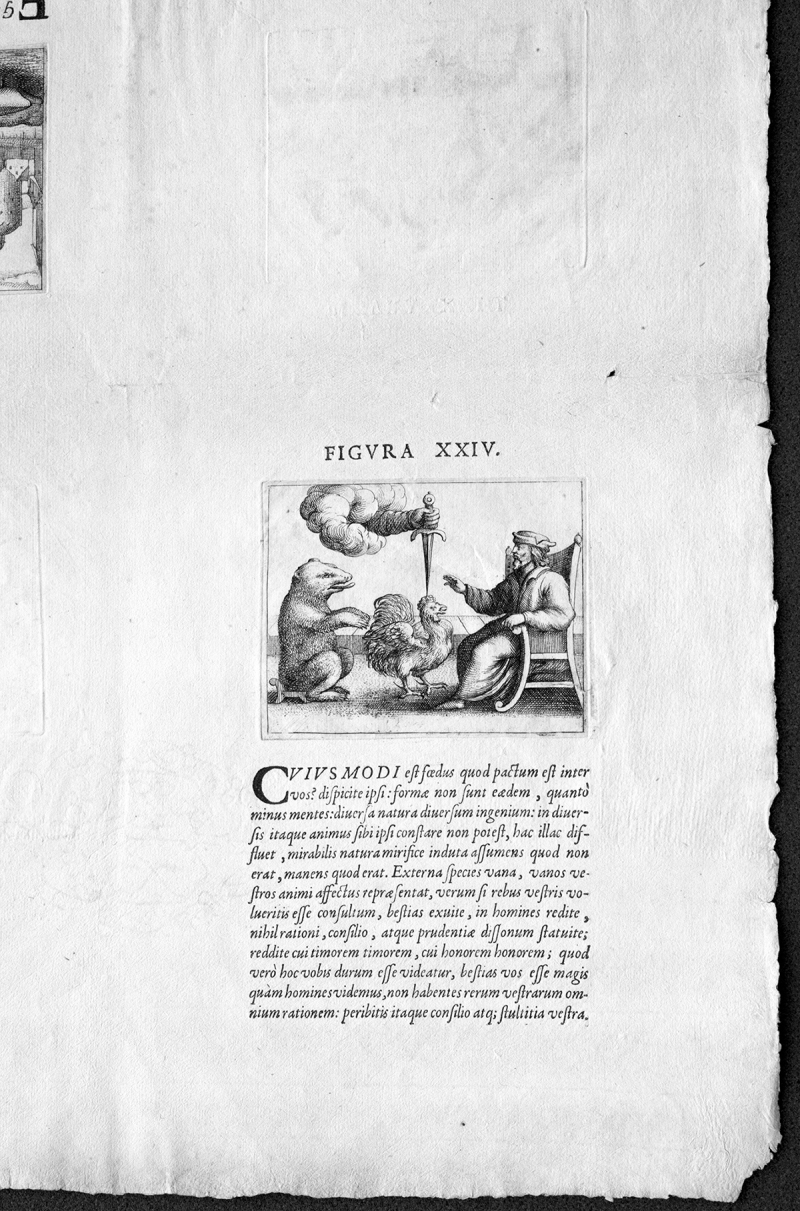
Text and engraving

The *Prognosticatio* does, however, present one additional complication. It is profusely illustrated with obscure, if interesting, mystical pictures. In this edition, they are copperplate engravings. The engravings were beautifully done and clearly added meaning to the text (for those in the know), but for the printer, they added another layer of complexity to the production. For technical reasons, the copperplates needed to go through a different sort of press for printing. Copperplate is an intaglio (recessed) process; the amount of pressure required to pull the ink from its grooves would destroy relief-made letterpress type. Earlier editions were illustrated with woodcuts, which did not present this problem but which were also inferior in quality to the engravings. For the printer, including engravings meant that each sheet of paper went through presses four times: once on each side for the image and once on each side for the text. Figure 2 shows both the text and engraving of a single page in close-up; the upside down plate of another page on the same sheet is just visible in the upper left corner.

It will never be known why this copy was left uncompleted. The most likely explanation is that it simply did not sell. Sheets could be bound at the time of sale, or folded and stitched without binding, or even sold loose and unfolded, with the purchaser taking responsibility for the finishing. However, someone must have thought the sheets worth keeping. There were many other uses to which waste sheets could be put by either the printer or the bookbinder.

The sheets now reside in a custom-made acid free box at NLM; they will never be bound. They are a reminder of the sheer physicality of an early printed book and all of the work involved in its creation. It was all handwork: the paper, the ink, the hand-cast type, the hand-engraved copperplates, the presswork, and the folding, sewing, and binding that never took place. Master craftsmen, journeymen, and apprentices working long days in dark, damp shops. All to make ten sheets that were never to be sold and never to be read.

